# P346: Evaluation of hygiene in hospital - about 50 services

**DOI:** 10.1186/2047-2994-2-S1-P346

**Published:** 2013-06-20

**Authors:** MJ Bitty, YB Coha, JD Seka, F Monan

**Affiliations:** 1 Direction of Public Hygiene, Ministry of Health and fight against aids, Abidjan, Côte d'Ivoire

## Introduction

This study was undertaken to assess the status of implementation of directives of Public Health in Côte d'Ivoire, to provide regional data see national hospital hygiene, medical waste management, injection safety, to make a plea to local authorities for ownership of hospital hygiene by health workers and local authorities and the community. For this purpose, we present the method of work, the results and comments arising from these.

## Objectives

To analyze hospital hygiene, injection safety and medical waste management services in 50 health districts located in Yopougon Aboisso Sassandra Daoukro Toumodi, Yamoussoukro, Seguela, Jacqueville Grand lahu, Côte d'Ivoire.

### Specific Objectives

Assess the organization of hospital hygiene, Analyze the safety of injections, Evaluate the management of medical waste, Measure the treatment of blood exposure accidents (AES).

## Methods

This is a retrospective study referred for analytical data collected in the departments of Jacqueville Grand Lahou and the health districts of Yopougon (CHU de Yopougon) Aboisso of Sassandra, Daoukro Toumodi, Yamoussoukro, and Séguéla. The collection of information about each service was done using a survey form created for this purpose. The data collected were analyzed using computer software STATISTICA version 7.1.

## Results

The organization of hospital hygiene is not satisfactory in the target services: the rate of implementation of the various parameters studied below 40%. It notes, however, 68% of health facilities targets, the existence of hospital hygiene committee. Hand hygiene is applied to 65% in Services. All health care workers wear gowns, but approximately 68% of them wear street clothes.

In general, the practice of safe injections is well respected although there are wide disparities between the health facilities. The medical waste management is a real problem, the rate of good management is less than 50%. It is absent in some health districts such as Séguéla in northern Côte d'Ivoire.

## Conclusion

Generally less than 50% of health facilities surveyed provide good support for blood exposure accidents.

## Disclosure of interest

None declared.

